# Cyclodextrin Derivatives as Promising Solubilizers to Enhance the Biological Activity of Rosmarinic Acid

**DOI:** 10.3390/pharmaceutics14102098

**Published:** 2022-09-30

**Authors:** Anna Stasiłowicz-Krzemień, Natalia Rosiak, Anita Płazińska, Wojciech Płaziński, Andrzej Miklaszewski, Ewa Tykarska, Judyta Cielecka-Piontek

**Affiliations:** 1 Department of Pharmacognosy, Faculty of Pharmacy, Poznan University of Medical Sciences, Rokietnicka 3, 60-806 Poznan, Poland; 2 Department of Biopharmacy, Faculty of Pharmacy, Medical University of Lublin, Chodzki 4a, 20-093 Lublin, Poland; 3 Jerzy Haber Institute of Catalysis and Surface Chemistry, Polish Academy of Sciences, Niezapominajek 8, 30-239 Krakow, Poland; 4 Institute of Materials Science and Engineering, Poznan University of Technology, Jana Pawla II 24, 61-138 Poznan, Poland; 5 Department of Chemical Technology of Drugs, Poznan University of Medical Sciences, Grunwaldzka 6, 60-780 Poznan, Poland

**Keywords:** rosmarinic acid, cyclodextrins, neurodegeneration, solubility, permeability, blood–brain barrier

## Abstract

Rosmarinic acid (RA) is a natural antioxidant with neuroprotective properties; however, its preventive and therapeutic use is limited due to its slight solubility and poor permeability. This study aimed to improve RA physicochemical properties by systems formation with cyclodextrins (CDs): hydroxypropyl-α-CD (HP-α-CD), HP-β-CD, and HP-γ-CD, which were prepared by the solvent evaporation (s.e.) method. The interactions between components were determined by X-ray powder diffraction (XRPD), differential scanning calorimetry (DSC) and Fourier Transform infrared spectroscopy (FTIR). The sites of interaction between RA and CDs were suggested as a result of in silico studies focused on assessing the interaction between molecules. The impact of amorphous systems formation on water solubility, dissolution rate, gastrointestinal (GIT) permeability, and biological activity was studied. RA solubility was increased from 5.869 mg/mL to 113.027 mg/mL, 179.840 mg/mL, and 194.354 mg/mL by systems formation with HP-α-CD, HP-β-CD, and HP-γ-CD, respectively. During apparent solubility studies, the systems provided an acceleration of RA dissolution. Poor RA GIT permeability at pH 4.5 and 5.8, determined by parallel artificial membrane permeability assay (PAMPA system), was increased; RA–HP-γ-CD s.e. indicated the greatest improvement (at pH 4.5 from *P_app_* 6.901 × 10^−7^ cm/s to 1.085 × 10^−6^ cm/s and at pH 5.8 from 5.019 × 10^−7^ cm/s to 9.680 × 10^−7^ cm/s). Antioxidant activity, which was determined by DPPH, ABTS, CUPRAC, and FRAP methods, was ameliorated by systems; the greatest results were obtained for RA–HP-γ-CD s.e. The inhibition of acetylcholinesterase (AChE) and butyrylcholinesterase (BChE) was increased from 36.876% for AChE and 13.68% for BChE to a maximum inhibition of the enzyme (plateau), and enabled reaching IC_50_ values for both enzymes by all systems. CDs are efficient excipients for improving RA physicochemical and biological properties. HP-γ-CD was the greatest one with potential for future food or dietary supplement applications.

## 1. Introduction

Rosmarinic acid (RA) is a natural phenolic acid in many plants, including herbs and spices such as rosemary, oregano, sage, thyme, and lemon balm. It is a significant antioxidant with anti-inflammatory, antidiabetic, neuroprotective, anticancer, antimicrobial, and antiviral properties [1,2,3,4]. Its pro-health properties have been studied through in vitro tests on enzymes and cell lines, as well as in vivo [5,6,7].

Its extraordinary preventive and therapeutic potential is hampered by its limited solubility. Most of the compounds of rosemary extract are assigned to the III or IV class of the Biopharmaceutics Classification System (BCS) due to ambiguous solubility and low permeability [8]. RA is believed to have slight solubility and low permeability, subsequently impacting low absorption. In the study on rats, RA bioavailability was assessed, ranging from 0.91% to 1.69%, and characterized by parameters such as rapid absorption, middle-speed elimination, and poor absolute bioavailability [9]. RA has low permeability; it shows intensive conjugation in Caco-2 cells after deconjugation by phase II xenobiotic-metabolizing enzymes in the intestine. Unfortunately, during studies, the presence of RA after the oral administration of *Melissa officinalis* extract containing 100 mg of RA in almost all healthy volunteers was not determined [10]. Moreover, the LLOQ and LLOD failed to detect RA in plasma samples for the majority of the volunteers after the administration of the *Thunbergia laurifolia* leaf extract [8].

One of the possibilities of improving the physicochemical parameters of RA is its systems formation with cyclodextrins (CDs). CDs are conical-shaped cyclic oligosaccharides derived from starch [11]. CDs form host–guest complexes in gas phase, solution, and in solid states with hydrophobic molecules by electrostatic, van der Waals, hydrogen bonding, and hydrophobic interactions, thus, enabling the improvement of the physicochemical properties of the guest substance, such as solubility, permeability, stability, and volatility [12,13].

So far, CDs have been used to improve the physicochemical parameters of many substances, including RA. RA complexes were previously formed with α-CD, β-CD, 2-hydroxypropyl-β-CD (HP-β-CD), 2-hydroxyethyl-β-CD (HE-β-CD), and methyl-β-CD (M-β-CD), and the impact on antioxidant activity was determined [14]. In another study, 2-HP-β-CD and AC-β-CD were used to impact photo-stability and antioxidant activity [15]. The influence of HP-β-CD and M-β-CD on RA antioxidant properties was also studied by Kleyton et al. [16]. To the authors’ greatest knowledge, HP-α-CD and HP-γ-CD complexes or systems with RA were not previously described. Moreover, the impact on neuroprotective activity has not yet been studied.

This study aimed to enhance the physicochemical properties and biological activity of RA with CDs by systems preparation by the solvent evaporation method. The interactions between RA and CDs were studied by X-ray powder diffraction (XRPD), differential scanning calorimetry (DSC), and attenuated total reflectance Fourier Transform infrared spectroscopy (ATR-FTIR), supported by in silico calculations. The impact of CDs on RA on 24-h water solubility, dissolution rate, and membrane permeability in the gastrointestinal tract (GIT) studied by parallel artificial membrane permeability assay (PAMPA system) was determined. The CDs’ influence on RA biological activity was also determined in terms of (i) antioxidant activity (studied in 2,2-diphenyl-1-picrylhydrazyl (DPPH), 2,2′-azino-bis(3-ethylbenzothiazoline-6-sulfonic acid) (ABTS), cupric reducing antioxidant capacity (CUPRAC), and ferric reducing antioxidant power assay (FRAP) methods); (ii) the inhibition of enzymes influencing the development of neurodegenerative diseases—acetylcholinesterase (AChE), butyrylcholinesterase (BChE), and tyrosinase.

## 2. Materials and Methods

### 2.1. Materials

RA (purity > 95%); 2-HP-α-CD (molar substitution 0.6, Mw~1.180); 2-HP-β-CD (molar substitution 0.8, Mw~1.460); and 2-HP-γ-CD (0.5–0.7 molar substitution, Mw~1.580) were purchased from Sigma-Aldrich (Poznan, Poland). Acetonitrile (high-performance liquid chromatography [HPLC] grade) was provided by Merck (Darmstadt, Germany), and other chemical reagents—including hydrochloric acid, dimethyl sulfoxide, sodium chloride, and potassium dihydrogen phosphate—were obtained from Avantor Performance Materials (Gliwice, Poland). Formic acid 98–100% was purchased from POCH (Gliwice, Poland). Prisma HT, GIT lipid solution, and acceptor sink buffer were supplied by Pion Inc (Forest Row, East Sussex, England).

### 2.2. Methods

#### 2.2.1. The Preparation of the Systems

RA systems with HP-α-CD, HP-β-CD, and HP-γ-CD in a mass ratio of 1:1 were prepared by the solvent evaporation method. RA and CDs HP-α-CD, HP-β-CD, and HP-γ-CD were dissolved in 70% (*v*/*v*) ethanol and evaporated using Rotavapor (BUCHI System, Flawil, Switzerland) at the temperature of 40 °C. The resulting systems were grounded in a mortar and stored with physical mixtures of the binary systems in a desiccator until further tests were performed.

#### 2.2.2. Identification of the systems of RA with Cyclodextrins

##### X-ray Powder Diffraction

The crystallic state of RA, HP-α-CD, HP-β-CD, HP-γ-CD, the state of their physical mixtures, and their systems were determined with the XRPD method. Diffraction patterns were measured on a PANalytical Empyrean diffractometer with CuKα radiation (1.54056 Å) at a tube voltage of 45 kV and a tube current of 40 mA. The angular range was 3° to 50° with a step size of 0.017° and a counting rate of 15 s/step. The OriginPro 8 software was used to analyze the acquired data [17].

#### Differential Scanning Calorimetry

The thermal analysis was performed using a DSC 214 Polyma differential scanning calorimeter (Netzsch, Selb, Germany). Samples of about 8–10 mg were placed in crimped aluminum pans with a small hole in the lid. The measurements were performed at a constant heating rate of 10° K·min^−1^ under a nitrogen atmosphere with a flow rate of 30 mL·min^−1^ in the range 25–200 °C.

#### Fourier Transform Infrared Spectroscopy

The (ATR-FTIR) spectra were recorded on an IRTracer-100 spectrophotometer (Kyoto, Kyoto Prefecture, Japan). The spectra were measured within the frequency range of 4000 and 400 cm^−1^ in the absorbance mode. The parameters of the apparatus were as follows: resolution, 4 cm^−1^; number of scans, 400; apodization, Happ-Genzel. The samples were placed on the ATR crystal and pressed against it whilst the ATR-FT-IR spectrum was scanned. The spectra of RA and CDs and the physical mixtures of the systems were also analyzed. The spectra were analyzed with the use of the OriginPro 8 software (OriginLab Corporation, Northampton, MA, USA).

#### Studies of Interactions of RA and Cyclodextrins

The RA molecule (guest) was drawn manually by using the Avogadro 1.1.1 software [18] and optimized within the UFF force field [19]. The carboxyl moiety was left deprotonated, as dictated by the experimental p*K_a_* value (ca. 3.6). The 2-HP-CD molecules (hosts) were prepared by relying on the available crystal structures of unfunctionalized α-, β-, and γ-CD (PDB: 4mtu, 5mk9 and 1d3c) and manually substituted by the 2-HP groups, according to the two alternative substitution patterns, by using Avogadro 1.1.1. Upon this modification, the six different host molecules were optimized using UFF force field.

From the known molar masses of the CD derivatives, it was deduced that the most probable number of 2-HP moieties present in one CD molecule is always close to the number of glucose residues composing a given CD molecule. Thus, the number of the covalently bound HP groups was assumed to be equal to either 6 (α-CD), 7 (β-CD), or 8 (γ-CD). However, the exact substitution pattern remains unknown; thus, the two alternative possibilities were considered: (1) 2-HP-CD^#^ containing all 2-HP groups substituted to the O_(2)_ hydroxyl oxygen atoms of CD (atom numbering in accordance with the IUPAC recommendations); (2) 2-HP-CD* containing all 2HP groups substituted to the O_(6)_ hydroxyl oxygen atoms of the hydroxymethyl groups present in CD. These two cases represent the two topologically limiting substitution patterns, placing all 2-HP moieties at opposite sides of the β-CD torus.

The guest–host docking was carried out by using the AutoDock Vina software [20]. The procedure of docking was performed within the cuboid region which covered the whole host molecule. The number of poses to generate was increased to 20. Apart from this, all the default procedures and algorithms implemented in AutoDock Vina were applied during docking. In addition to the flexibility of the guest molecules, the rotation of all the exocyclic groups in the host molecules was also allowed.

#### 2.2.3. Chromatographic Studies of Changes of RA Concentrations

**Figure 1:**
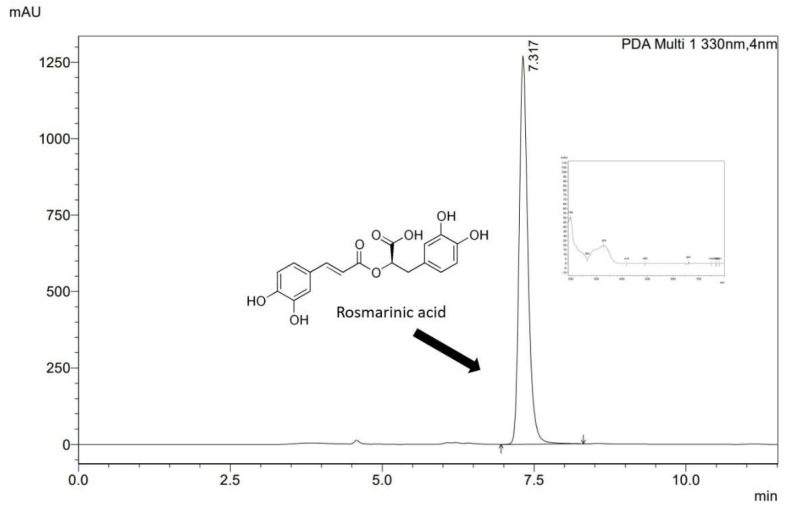
The chromatogram of RA.

The samples collected from the in vitro solubility, apparent solubility, and permeability studies were analyzed using HPLC with the diode array detector method (Shimadzu Corp., Kyoto, Japan). The RA determination was performed via a validated method using a stationary phase ReproSil-Pur Basic-C18 column (250 mm × 4.6 mm; 5 µm). The mobile phase consisted of 0.1% formic acid and acetonitrile (70:30, *v*/*v*). The flow rate of the mobile phase was set at 1.0 mL/min and the column temperature was 30 °C. The injection volume was 10.0 µL, and the detection wavelength was set at 330 nm. The analysis time was 11.5 min, and the retention time of RA was approximately 7.3 min (Figure 1). The results were obtained and processed by LabSolutions LC software (Shimadzu Corp., Kyoto, Japan).

#### 2.2.4. The Solubility Studies of RA

In this assay, excess amounts of RA and the systems were placed in glass vials, and 5 mL of distilled water was added. The samples were incubated for 24 h at 298.15 K at a constant speed of 75 rotations per minute (rpm) using a laboratory incubator MaxQ 4450 (Thermo Scientific, Waltham, MA, USA). Subsequently, the suspensions were filtered through 0.22 μm and analyzed by the HPLC method. All measurements were performed in triplicate.

#### 2.2.5. The Dissolution Studies of RA

The dissolution rate was determined in the paddle apparatus (Agilent Technologies, Santa Clara, CA, USA). RA (10.0 mg) and the systems (containing 10.0 mg of RA) were weighed into gelatin capsules and implemented into springs for floating prevention. The test was carried out in sink conditions for 180 min at a pH of 4.5 and 5.8, simulating the fed gastric and intestinal environments [21,22], as RA disintegrates at pH 1.2 [22,23,24]. The vessels were filled with 500 mL of media at the temperature set at 310.15 K and the rotation speed of 50 rpm. At specified time points, 2.0 mL samples were withdrawn and instantly substituted with an equivalent volume of the temperature-equilibrated fresh medium. Thereupon, the samples were filtered through a 0.22 μm membrane filter and analyzed by HPLC. The differences and similarities between the profiles were assessed by the two-factor values, *f*_1_, and *f*_2_, introduced by Moore and Flanner [22] with the use of the following equations:

(1) $$f_{1}=\frac{\sum_{j=1}^{n}\left|R_{j}-T_{j}\right|}{\sum_{j=1}^{n}R_{j}}$$

(2) $$f_{2}=50\times \log\left({\left(1+\left(\frac{1}{n}\right)\overset{n}{\underset{j=1}{\sum }}{\left|R_{j}-T_{j}\right|}^{2}\right)}^{-\frac{1}{2}}\times 100\right)$$

where *n* is the number of time points, Rj is the percentage of the reference dissolved substance in the medium, *Tj* is the percentage of the dissolved tested substance, and t is the time point. Dissolution profiles are described as similar when the f_1_ value is close to 0, or f_2_ is close to 100 (between 50 and 100) [25].

#### 2.2.6. Membrane Permeability of RA

The passive permeability through the biological membranes of RA was determined using the parallel artificial membrane permeability assay (PAMPA) models. The permeability study was assessed in the gastrointestinal (GIT) (pH 4.5 and 5.8) and blood–brain barrier (BBB) (pH 7.4) models. The study was performed in two 96-well microfilter plates, assigned as a donor (at the bottom) and acceptor (at the top) chambers, separated by a 120 μm-thick microfilter disc coated with a 20% (*w*/*v*) dodecane solution of a lecithin mixture (Pion Inc., Billerica, MA, USA). The RA and RA systems samples were dissolved in dimethyl sulfoxide (DMSO) and placed in donor solutions which were adjusted to pH ≈ 4.5 and 5.8 for GIT application and to pH ≈ 7.4 for BBB. Bearing in mind that CDs do not leave the GIT, the BBB permeability was only studied for RA. The plates were incubated in a humidity-saturated atmosphere with the temperature set at 310.15 K for 3 h for the GIT model and 4 h for the BBB assay. The plates were later separated; subsequently, the concentrations of RA were determined using the HPLC-DAD method. The *P_app_* was calculated using the equations:

(3) $$P_{app}=\frac{-ln\left(1-\frac{C_{A}}{C_{equilibrium}}\right)}{S\times \left(\frac{1}{V_{D}}+\frac{1}{V_{A}}\right)\times t}$$

(4) $$C_{equilibrium}=\frac{C_{D}\times V_{D}+C_{A}\times V_{A}}{V_{D}+V_{A}}$$

where *V_D_*—donor volume, *V_A_*—acceptor volume, *C_equilibrium_*—equilibrium concentration $C_{equilibrium}=\frac{C_{D}\times V_{D}+C_{A}\times V_{A}}{V_{D}+V_{A}}$, *S*—membrane area, and *t*—incubation time (in seconds).

Compounds with a *P_app_* in the GIT model below 0.1 × 10^−6^ cm/s are described as poorly permeable; substances with 0.1 × 10^−6^ cm/s ≤ *P_app_* < 1 × 10^−6^ cm/s are classified as mediocre permeable; and compounds found as highly permeable have a *P_app_* ≥ 1 × 10^−6^ cm/s [26]. Compounds whose *P_app_* in the BBB model is < 2.0 × 10^−6^ cm/s are classified as poorly permeable; compounds with questionable permeability have *P_app_* values in the range of 2.0 to 4.0 × 10^−6^ cm/s; and substances with high permeability have a *P_app_* value at the level > 4.0 × 10^−6^ cm/s [27].

#### 2.2.7. Biological Activity of RA

#### Antioxidant Activity of RA

Antioxidant activity was determined using 2,2-diphenyl-1-picrylhydrazyl (DPPH); 2,2′-azino-bis(3-ethylbenzothiazoline-6-sulfonic acid) (ABTS); cupric reducing antioxidant capacity (CUPRAC); and ferric reducing antioxidant power assay (FRAP) methods. CDs do not enter the bloodstream. Thus, the antioxidant activity of the systems was performed to evaluate if the excipients impact the antioxidant activity in addition to the influence of solubility enhancement within GIT.

The reaction between the methanol solution of DPPH (0.2 mM) and RA and the systems was measured spectrophotometrically [17]. The ascending concentrations of RA and RA in the systems from 10.0 μg/mL to 1.0 mg/mL (25.0 µL) were mixed with the DPPH solution (175.0 µL) on a 96-well plate, which was subsequently shaken in dark conditions for 30 min at room temperature and measured on a plate reader (Multiskan GO, Thermo Fisher Scientific, Waltham, MA, USA) at 517 nm. Simultaneously, the absorbance (A) was measured for the blank (mixture of DPPH solution and DPPH) at 517 nm. The inhibition of DPPH radicals by the tested samples was calculated using the following formula:

(5) $$A=\frac{A_{o\;}-A_{i}}{A_{o}}\times 100\%$$

where *A_o_* is the absorbance of the control sample and *A_i_* is the absorbance of the test sample. Each measurement was repeated 6 times. Trolox was used as a reference. IC_50_ values, which determine the concentration of a substance that inhibits DPPH formation by 50%, were determined by a linear regression analysis.

Another method for determining antioxidant activity is the ABTS modified method, which is performed according to Re et al. [28]. Green cation radicals are produced by the loss of electrons by the nitrogen atoms of ABTS caused by potassium persulfate. After introducing the preformed radical cation into the antioxidant, the ABTS radical cation is reduced back to its colorless neutral form. RA concentrations for the assay were prepared in 50% DMSO ranging from 10.0 μg/mL to 1.0 mg/mL. Equal concentrations of RA in the systems were prepared. Subsequently, 10.0 μL of the dilutions of RA and the systems and 200.0 μL of ABTS^•+^ solution were pipetted to 96-well plates. Later, the plates were incubated for 10 min at room temperature while shaking. After incubation, the absorbance values were measured at λ = 734 nm. Trolox was used as a standard. The inhibition of ABTS^•+^ by RA and the systems was calculated using the following formula:

(6) $$\mathrm{ABTS}\;\mathrm{scavenging}\;\mathrm{activity}\;(\%)=\frac{A_{0}-A_{1}}{A_{0}}\times 100\%$$

where:

*A*_0_—the absorbance of the control;

*A*_1_—the absorbance of the sample.

CUPRAC was another technique used to evaluate the antioxidant potential of RA, according to Apak et al. (with modifications) [29]. During assay, neocuproine and copper ion (II) complex interacted with compounds to determine their antioxidant potential. Phenolic groups of polyphenols oxidized to quinones, and the neocuproine and copper (II) ion complex (bluish) were reduced to the neocuproine and copper (I) ion complex (yellow). Equal volumes of acetate buffer at a pH of 7.0, a 7.5 mM ethanolic 96% solution of neocuproine, and a 10 mM solution of CuCl_2_·H_2_O were mixed to prepare the CUPRAC reagent. The RA concentrations for the method were prepared in the range of 1.0 μg/mL to 1.0 mg/mL in 50% DMSO, for the systems, respectively. Next, 50.0 µL of the RA and the systems solutions, and 150.0 µL of the CUPRAC reagent were applied to a 96-well plate, incubated with shaking at room temperature, and protected from light for 30 min. Later, the absorbance was measured at the wavelength set at 450 nm (Multiskan GO, Thermo Fisher Scientific, Waltham, MA, USA). Trolox was used as a standard reference. The IC_0.5_ value corresponds to the RA concentration indicating 0.5 absorbance, which was calculated by linear regression.

The FRAP technique is based on the reduction of colorless Fe^3+^ ion to Fe^2+^ with the simultaneous formation of a dark blue complex with 2,4,6-tris(2-pyridyl)-1,3,5-triazine (TPTZ) [30]. A total of 25.0 µL of RA or the systems was added to the 96-well plate with the FRAP mixture (25 mL acetate buffer, 2.5 mL TPTZ solution, and 2.5 mL of FeCl_3_·6H_2_O solution) and incubated in the dark for 30 min at 37 °C. Later, the absorbance was measured at the wavelength λ = 593 nm. (Multiskan GO, Thermo Fisher Scientific, Waltham, MA, USA). Six replicates were used in the analysis. Trolox was used as a standard reference. The IC_0.5_ value corresponds to the RA concentration indicating 0.5 absorbance, which was calculated similarly as in the CUPRAC assay.

#### Inhibition of Enzymes by RA influencing the Development of Neurodegenerative Diseases

The inhibition of AChE and BChE was performed using a spectrometric Ellman et al. modified assay [31]. This technique requires artificial substrates (thiocholine esters). Thiocholine is liberated during the enzymatic reactions with 5,5′-dithio-bis-(2-nitrobenzoic) acid (DTNB), and the 3-carboxy-4-nitrothiolate anion (TNB anion) is formed.

The enzyme activity is measured spectrophotometrically according to the increase in the thiocholine color in a 96-well plate. The RA concentrations for the assay were prepared in the range of 2.5 mg/mL to 25.0 mg/mL in DMSO. The wells contained 60.0 μL of 0.05 M Tris-HCl buffer, with a pH of 8.0, 20.0 μL of test solution, and 30.0 μL of AChE/BChE solution at a concentration of 0.2 U/mL. These were incubated while shaking for 5 min at room temperature. Next, 30.0 μL of 1.5 mM acetylthiocholine iodide (ATCI)/butyrylthiocholine iodide (BTCI) solution and 125.0 μL of 0.3 mM DTNB solution were added to the well and incubated in the same conditions for 20 min. Blanks for the test sample (the reaction mixture was stripped of the enzyme, the volume of Tris-HCl buffer was elevated); the control sample (solvent was pipetted instead of the test sample); and the blank for the control sample (the reaction mixture of the control sample was depleted of the enzyme (the volume of Tris-HCl buffer was elevated)) were also prepared. Galantamine was used as a reference. The measurements were performed at 405 nm. The percentage of the inhibition of AChE and BChE by the samples was calculated according to the equation:

(7) $$\mathrm{AChE}/\mathrm{BChE}\;\mathrm{inhibition}\;(\%)=\frac{1-\left(A_{1}-A_{1b}\right)}{\left(A_{0}-A_{0b}\right)}\times 100\%$$

where:

*A*_1_—the absorbance of the test sample;

*A*_1*b*_—the absorbance of the blank of the test sample;

*A*_0_—the absorbance of the control;

*A*_0*b*_—the absorbance of the blank of the control.

The tyrosinase inhibition assay uses L-DOPA, an amino acid used to replenish dopamine deficiencies, as a substrate for the tyrosinase enzyme. Tyrosinase catalyzes the metabolism of tyrosine to L-DOPA and L-DOPA to dopaquinone. The inhibition of tyrosinase causes a decrease in the degradation of L-DOPA, the precursor of dopamine, which is deficient in Parkinson’s disease patients. The reduction in the color intensity of the solution due to the inhibition of enzyme activity is the basis of the assay [32]. The inhibitor blocks L-DOPA access to the tyrosinase active site, which prevents the reaction from proceeding. In this assay, RA was dissolved in 50% DMSO in ascending concentrations from 50.0 μg/mL to 2.5 mg/mL. The test sample contained 75.0 μL of 0.1 M phosphate buffer with a pH of 6.8, 25.0 μL of test solution, and 50.0 μL of enzyme solution (192 U/mL). The samples were incubated while shaking at room temperature for 10 min. Next, 50 μL 2.0 mM L-DOPA was pipetted to the wells and incubated in the same conditions for another 20 min. Blanks for the test sample (the reaction mixture was depleted of the enzyme, the volume of phosphate buffer was increased); the control sample (solvent was introduced instead of the test sample); and the blank for the control sample (the reaction mixture of the control sample was stripped of the enzyme (the volume of phosphate buffer was increased)) were also prepared. The absorbance of the test samples was measured at a wavelength of 475 nm. Azelaic acid was used as a standard. The percentage inhibition of the tyrosinase by the samples was calculated using the equation:

(8) $$\mathrm{Tyrosinase}\;\mathrm{inhibition}\;(\%)=\frac{1-\left(A_{1}-A_{1b}\right)}{\left(A_{0}-A_{0b}\right)}\times 100\%$$

*A*_1_—the absorbance of the test sample;

*A*_1*b*_—the absorbance of the blank of the test sample;

*A*_0_—the absorbance of the control;

*A*_0*b*_—the absorbance of the blank of the control.

### 2.2.8. Statistical Analysis

Statistica 13.3 software (StatSoft Poland, Krakow, Poland) was used for the statistical analysis. Data are presented as mean values ± standard deviations. Experimental data were analyzed using the Shapiro–Wilk test to determine the normality of each distribution, and the Levene’s test assessed the equality of variances. Statistical significance was determined using a one-way analysis of variance (ANOVA) followed by the Bonferroni post hoc test (to compare the experimental results for RA and the systems). Differences were considered significant at *p* < 0.05.

## 3. Results

### 3.1. The Preparation of the Systems of RA with Cyclodextrins

This study shows how to obtain the systems of RA with CDs by the solvent evaporation method in order to improve its solubility. In the literature, there are combinations of RA with various excipients such as CDs and polymers, e.g., hydroxypropyl methylcellulose, microcrystalline cellulose, lactose monohydrate, polyvinylpyrrolidone, polysulfated propylene-polyethylene glycol to evaluate compatibility, the influence on antioxidant activity, anti-inflammatory activity, stability, or bioavailability [14,15,33,34]. Physical mixtures of polymers and RA were prepared for compatibility determination, while electrospinning and freeze-drying techniques were used for obtaining the systems [16,33,35]. To the best of the authors’ knowledge, HP-α-CD, HP-γ-CD, and the solvent evaporation method have not yet been used in the literature to influence the physicochemical parameters of RA and its neuroprotective potential.

### 3.2. X-ray Powder Diffraction

**Figure 2:**
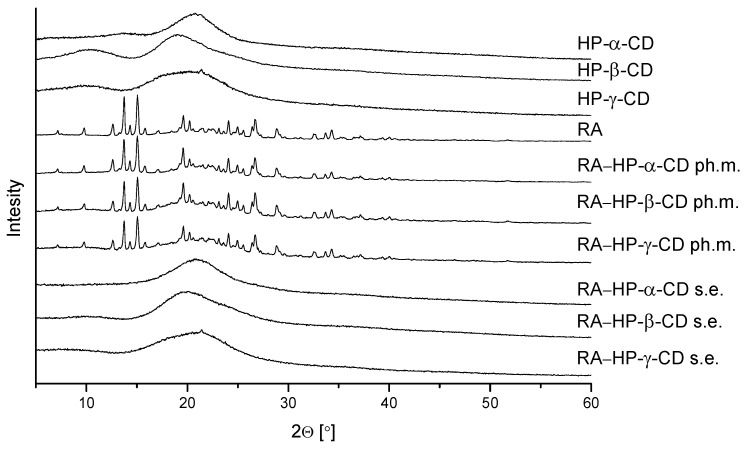
The XRPD diffraction patterns of HP-α-CD, HP-β-CD, HP-γ-CD, RA, physical mixtures: RA–HP-α-CD ph.m., RA–HP-β-CD ph.m., RA–HP-γ-CD ph.m., and systems obtained by the solvent evaporation method (s.e.): RA–HP-α-CD s.e., RA–HP-β-CD s.e., RA–HP-γ-CD s.e.

An XRPD study was carried out to determine the crystallinity of RA, RA, and CDs systems’ and physical mixtures (Figure 2). Pure RA has a crystalline structure. Its diffractogram contains sharp reflexes in the range of 2θ from 10° to 35°. Diffractograms of RA physical mixtures with CDs do not show any decrease in or disappearance of these reflexes, thus, remaining in the crystalline state. However, the diffractograms of the RA systems obtained by solvent evaporation are similar to the diffractograms of CDs. The RA reflexes are imperceptible. Therefore, it is possible to suggest the formation of amorphous dispersions of RA with CDs: HP-α-CD, HP-β-CD, and HP-γ-CD.

### 3.3. Differential Scanning Calorimetry

**Figure 3:**
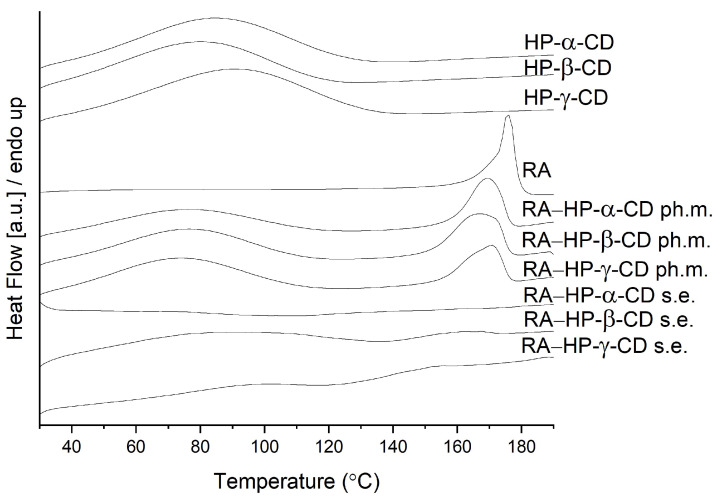
The DSC analysis of HP-α-CD, HP-β-CD, HP-γ-CD, RA, physical mixtures: RA–HP-α-CD ph.m., RA–HP-β-CD ph.m., RA–HP-γ-CD ph.m., and systems obtained by the solvent evaporation method (s.e.): RA–HP-α-CD s.e., RA–HP-β-CD s.e., RA–HP-γ-CD s.e.

Differential scanning calorimetry (DSC) was used to recognize the thermal properties of the tested systems. Each measurement involved one heating cycle. The thermograms are shown in Figure 3. The DSC curves of RA showed sharp endothermic peaks at 175.8 °C, which are attributed to the melting points [36]. The DSC thermogram of HP-α-CD, HP-β-CD, and HP-γ-CD revealed an endothermic peak at 83.5 °C, 79.6 °C, and 90.5 °C, respectively, which corresponds to the release of water molecules [37]. The physical mixtures were found to contain two distinguishable peaks: one around 76 °C and one in 169.3 °C (RA–HP-α-CD); 76.7 °C and in 167.0 °C (RA–HP-β-CD); and 73.6 °C and in 170.6 °C (RA–HP-γ-CD). The melting endotherm disappeared in all RA–cyclodextrin systems, which indicated the loss of the crystallinity of RA, as well as the formation of intermolecular interactions between RA and cyclodextrin.

### 3.4. Fourier Transform Infrared Spectroscopy

**Figure 4:**
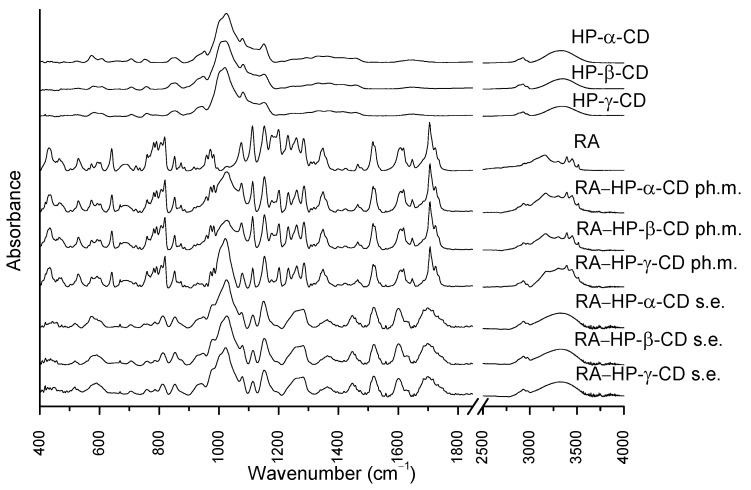
The FT-IR spectra of HP-α-CD, HP-β-CD, HP-γ-CD, RA, physical mixtures: RA–HP-α-CD ph.m., RA–HP-β-CD ph.m., RA–HP-γ-CD ph.m., and systems obtained by the solvent evaporation method (s.e.): RA–HP-α-CD s.e., RA–HP-β-CD s.e., RA–HP-γ-CD s.e.

The RA FTIR spectrum (Figure 4.) comprises characteristic peaks related to its structure, such as 3454 cm^−1^ and 3396 cm^−1^ connected with O-H stretching vibrations, and 3168 cm^−1^, which is associated with the stretching vibration of the C-H bond. Bands at 1724 cm^−1^ and 1705 cm^−1^ correspond to C=O stretching vibrations, while the peak at 1647 cm^−1^ is related to C=C stretching vibrations. In the wavenumber of 1464 cm^−1^ the peak is associated with CH_2_ bending vibrations. The peak at 1284 cm^−1^ is related to aromatic C-H bending vibrations, whereas the band at 1077 cm^−1^ corresponds to aromatic C-H bending vibrations and C=O stretching vibrations. The bands within the wavelength from 850 to 688 cm^−1^ are associated with aromatic C-H vibrations.

The spectra of HP-α-CD, HP-β-CD, and HP-γ-CD seem similar, yet there are slight changes in the intensities of the peaks. The CD spectra comprise intense broad peaks at 3354 cm^−1^ and bands within the range 1000–1200 cm^−1^ associated with the symmetric and antisymmetric O-H stretching vibrations of the molecules. Between the range 2500 and 3000 cm^−1^ are peaks associated with the symmetric and antisymmetric stretching vibrations of the C-H bond. The band at 1646 cm^−1^ is related to H–O–H bending vibrations. The zone from 1200 cm^−1^ to 1500 cm^−1^ contains peaks related to the CH_2_ and O-H bonds wagging vibrations.

The RA bands at 3454 cm^−1^, 3396 cm^−1^, and 3168 cm^−1^ corresponding to O-H and C-H stretching vibrations were merged with the broad O-H peak of CDs on the spectra of the systems. These changes might not unequivocally indicate the formation of an interaction between RA and CDs [38]. However, the same peaks in the spectra of the physical mixtures with HP-α-CD, HP-β-CD, and HP-γ-CD were easily differentiated. Moreover, the CDs broad band was shifted from 3354 cm^−1^ to 3346 cm^−1^ [39]. The RA peaks at 1724 cm^−1^ and 1705 cm^−1^ connected with C=O stretching vibrations were present in physical mixtures, while in the systems, they were broadened and reduced. The RA band at 1077 cm^−1^ was decreased in the systems spectra. Considering all shifts in bands in the spectra and implementing theoretical calculations, there was a possible host–guest system formation between CDs and RA by hydrogen bond formation.

### 3.5. Studies of RA Interactions with Cyclodextrins

**Figure 5:**
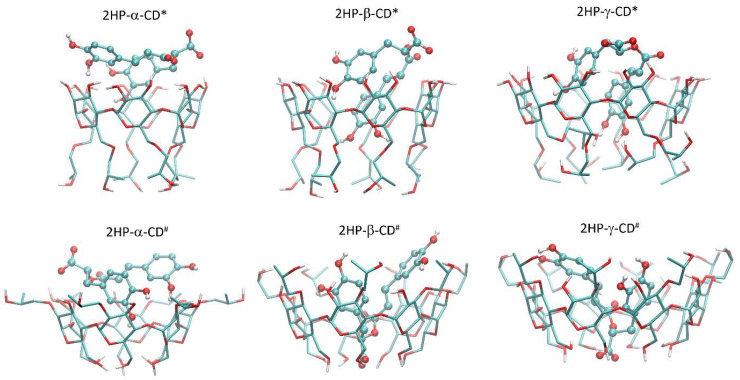
The graphical illustration of the RA–CD systems identified during the docking study. The shown structures are representative of the given type of host molecule. The guest (RA) molecule is shown in a ball-and-stick representation, whereas the host (various derivatives of CDs) molecules are presented in stick representation. Aliphatic hydrogen atoms are omitted for clarity. #: 2-HP-CD# containing all 2-HP groups substituted to the O(2) hydroxyl oxygen atoms of CD (atom numbering in accordance with the IUPAC recommendations); *: 2-HP-CD* containing all 2HP groups substituted to the O(6) hydroxyl oxygen atoms of the hy-droxymethyl groups present in CD.

Docking simulations allowed for the identification of various guest–host systems that contribute to the systems formation. Apart from a very general pattern of systems formation (guest molecules located in the center of a binding cavity formed by the inner channel of the CD molecule) common for all systems, there exist a series of substantial differences dependent on both the CD type and its substitution pattern. Moreover, the large number of distinct molecular arrangements exhibiting nearly the same binding energy value was detected. This can be interpreted in terms of the high flexibility of the whole molecular system and, especially, the significant mobility of the bound guest molecule within the CD channel. Below, the main features of the guest–host binding patterns are described for each of the possible cases. Those features were determined on the basis of an analysis of systems characterized by very similar binding energy values (within the threshold of ca. 0.4 kcal/mol). The graphical illustration of the results is given in Figure 5.

2-HP-α-CD*-containing systems: The guest molecule does not fully enter the CD inner channel but rather interacts with the exocyclic hydroxyl groups of CD (via hydrogen bonding with a contribution of both hydroxyl, ester, and carboxyl groups). Some scarce, CH–π attractive interactions are possible, involving dihydroxyphenyl groups of the guest molecule and the inner surface of glucose rings.

2-HP-α-CD^#^-containing systems: As in the case of 2-HP-α-CD*, but now the area of the guest–host contact is increased by the presence of the 2HP moieties substituted to the hydroxyl groups. Again, the driving force for binding is provided by hydrogen bonding and CH–π interactions, currently also involving the 2-HP groups.

2-HP-β-CD*-containing systems: The guest molecule is partially immersed in the inner channel of the host. One of the dihydroxyphenyl moieties creates the intensive, CH-π-driven contacts with the inner surface of the channel, whereas the remaining part of the guest molecule interacts via hydrogen bonding with the hydroxyl groups of CD. The carboxyl and ether groups exhibit very limited contact with CD.

2-HP-β-CD^#^-containing systems: The guest molecule is nearly fully immersed within the CD inner channel. Both dihydroxyphenyl groups create the network of CH–π interactions with either the inner surface of glucose rings or the aliphatic regions of the 2-HP groups. Additional intensive hydrogen bonds with CD involving both dihydroxyphenyl are also present. The carboxyl moiety of the guest molecule is located deep within the CD channel in the proximity of the hydromethyl groups and can interact with them via hydrogen bonding.

2-HP-γ-CD*-containing systems: A binding pattern similar to that exhibited by 2-HP-β-CD*. The guest molecule interacts with the inner channel of the host by one of the dihydroxyphenyl moieties and by exploiting both the CH-π-mechanism and hydrogen bonding. The remaining part of the guest molecule interacts via hydrogen bonding with the hydroxyl groups of CD, whereas the carboxyl and ether groups exhibit very limited contact with CD.

2-HP-γ-CD^#^-containing systems: A binding pattern similar to that exhibited by 2-HP-β-CD^#^. The guest molecule is nearly fully immersed within the inner CD channel. Both dihydroxyphenyl groups create the network of CH–π interactions with both the inner surface of glucose rings and the aliphatic regions of the 2HP groups. Moreover, hydrogen bonding between 2-HP groups and the ring oxygens of the CD and hydroxyl groups of the guest appears. Again, the carboxyl moiety is located deep within the CD channel and creates hydrogen bonds with the hydroxymethyl groups of CD.

The above-described patterns of the guest–host interactions are fairly well-maintained across the complete set of studied guest–host structures obtained for a given type of host molecule. However, as mentioned above, a number of minor differences between observed contacts were also identified. Those differences mainly involve the most flexible groups of guest molecules, i.e., 2-HP, hydroxymethyl, and hydroxyl moieties. The interactions with and within such groups (involving intra- and intermolecular hydrogen bonding) are not necessarily maintained across the whole set of low-energy structures. A relatively low scatter in determined binding energies accompanied by notable structural differences suggests the high flexibility of the whole molecular systems, independently of the considered substitution pattern.

The binding energies collected in Table 1 confirm that the most intensive guest–host contacts (observed in the cases of 2-HP-β-CD^#^ and 2-HP-γ-CD^#^) are correlated with the most favorable interaction energies. Nevertheless, the process of systems formation is expected to always be a favorable one, as the accompanying energy values exceed ca. 5 kcal/mol. From the perspective of binding strength, the affinity of RA to various CDs can be ordered as follows: 2-HP-α-CD < 2-HP-β-CD ~ 2-HP-γ-CD. Moreover, the substitution pattern # (i.e., the 2-HP moieties attached to hydroxyl groups) always corresponds to more favorable binding energies in comparison to the alternative pattern. This is in line with a more intensive network of guest–host contacts detected in the former case and associated with the presence of 2-HP moieties at the broader entrance to the CD inner channel. This finding emphasizes the significance of the presence of 2-HP groups in the structure of the host molecule on its systems formation ability. As shown here, the place of the substitution of these groups is also an essential factor influencing the features of host molecules. Based on the current case, it can be stated that the systems formation ability is improved when the 2-HP groups are substituted at hydroxyl moieties, i.e., at the broader entrance to the inner channel of CD.

Finally, let us note that the above analysis, considering the two alternative substitution patterns of the 2-HP groups, has no aim to identify the most favorable binding mode and to discriminate between those two possibilities. As the real substitution pattern is unknown, any of these two above possibilities can occur, as well as their combination or yet another substitution pattern (e.g., an intermediate between limiting cases discussed here). A series of similarities between the binding parameters discussed here and determined for different CD derivatives allow us to assume that the obtained binding characteristics are a fair representation of some more diverse systems containing 2-HP substituents.

### 3.6. The Solubility Study of RA

**Figure 6:**
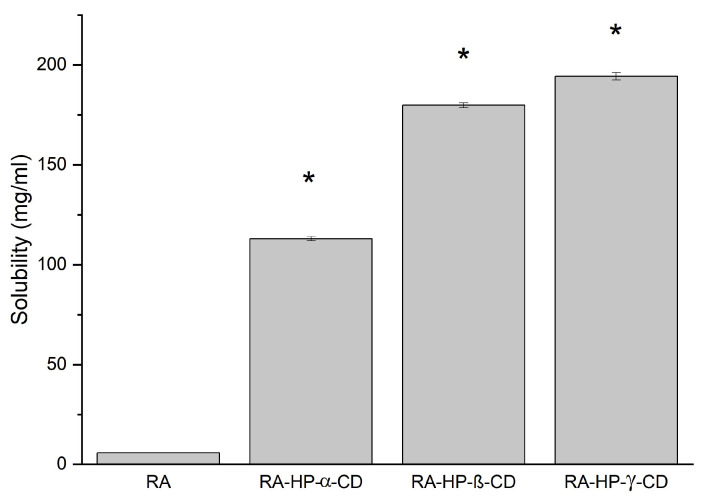
Solubility of RA and RA in binary systems prepared by the solvent evaporation method: RA–HP-α-CD s.e., RA–HP-β-CD s.e., RA–HP-γ-CD s.e. (*) indicates statistically significant differences, *p* < 0.05.

The water solubility of the RA was assessed as 5.869 ± 0.140 mg/mL (Figure 6). The preparation of the systems with CDs by the solvent evaporation method significantly increased the solubility of the phenolic acid. The greatest enhancement was observed for RA–HP-β-CD s.e.—179.840 ± 1.279 mg/mL, RA–HP-γ-CD s.e.—194.354 ± 1.847 mg/mL. The interaction between the RA into the HP-γ-CD cavity resulted in a 33-fold increase in solubility.

RA [40] is usually described as slightly/poorly soluble [8]. There are few studies and literature reports on RA solubility because it is a biologically active substance, often studied as an extract component. Its water solubility was estimated in silico as 0.041 g/L (ALOGPS) [8]. The aqueous solubility of the RA in pH 1.2 was determined as close to 5 mM [16], while at pH 7.2 in PBS, the solubility was approx. 15 mg/mL [41]. Due to its structure, it is more soluble in most organic solvents (approx. 25 mg/mL) [35]; subsequently, it is more soluble in mixtures of alcohols and water than in pure water [42].

CDs are widely used as excipients to increase the physicochemical properties of both synthetic compounds and natural substances, e.g., HP CD derivatives have a great potential to improve solubility. Depending on the compound to be incorporated into the cavity of the CD, the effect of different CDs on the solubility of the molecule can vary; for example, in the case of curcumin, HP-β-CD caused an increase in solubility ~57 times, while better results were obtained for HP-γ-CD, which caused an increase in solubility ~123 times [43,44]. In another study, HP-α-CD, HP-β-CD, and HP-γ-CD impacted the solubility of phenolic monoterpenes thymol and carvacrol [45]. For both substances, the most efficient increase in solubility was caused by the influence of HP-β-CD. The lowest rise in solubility was observed for HP-α-CD. Perillaldehyde inclusion complex nanofiber with HP-γ-CD obtained by electrospinning caused an increase in aqueous solubility and improved physicochemical properties [46]. Cuminaldehyde and isoeugenol inclusion complexes with M-β-CD prepared in aqueous solution and in a solid state via the ultrasonication method increased the solubility ten and twelve times, respectively [47]. In studies on electrospinning, the water solubility of pyrimethanil was enhanced due to the inclusion of compound nanofibers preparation with HP-β-CD, whilst the solubility of difenoconazole was increased about five times in a 10 mM solution of HP-β-CD [48,49].

### 3.7. The Apparent Solubility Study of RA

**Figure 7:**
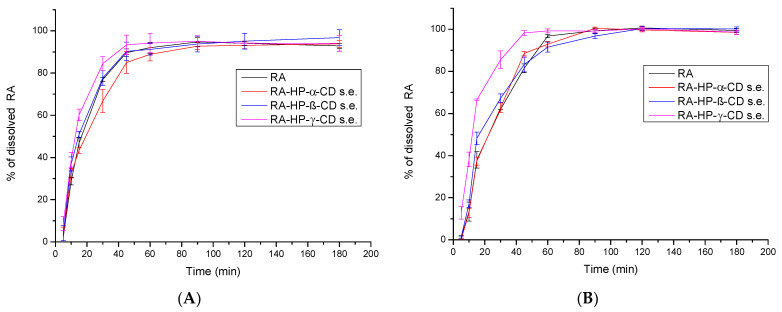
Apparent solubility of RA and RA in binary systems prepared by the solvent evaporation method: RA–HP-α-CD s.e., RA–HP-β-CD s.e., RA–HP-γ-CD s.e. in a pH of 4.5 (**A**) and in a pH of 5.8 (**B**).

In a dissolution rate test in a pH of 4.5 (Figure 7A), RA reached 46.94 ± 2.32% in 15 min and 89.97 ± 1.79% in 45 min, with a maximum dissolution rate of 94.74 ± 2.13 reached in 90 min. For RA in the RA–HP-β-CD s.e. system, differences in dissolution profiles were minimal, while RA–HP-α-CD s.e. caused a slight decrease in dissolution. RA in RA–HP-γ-CD s.e. dissolved at 60.20 ± 2.76% in 15 min; thus, 15% more RA was dissolved due to the formation of the system with HP-γ-CD in just 15 min. At 30 min, 84.52 ± 3.18% RA in the system was dissolved. While the apparent solubility was accelerated, the overall dissolution level did not increase significantly.

At pH 5.8 (Figure 7B), RA dissolved at 37.97 ± 1.04% in 15 min; in 45 min, it reached 81.48 ± 2.10%. The maximum dissolution level was reached in the ninetieth minute at 99.35 ± 1.27% and remained constant. The dissolution profile of RA in the RA–HP-α-CD s.e. system was almost identical to RA itself. Interactions with HP-β-CD accelerated the dissolution—at 15 min 48.27 ± 2.93%; however, these differences were not statistically significant. RA in the RA–HP-α-CD s.e system reached 66.53 ± 0.43% in the fifteenth minute, and 98.277 ± 1.19% in the sixtieth minute remained constant. The dissolution profile of RA in this system is statistically different from the pure RA.

### 3.8. The Permeability Study of RA

**Figure 8:**
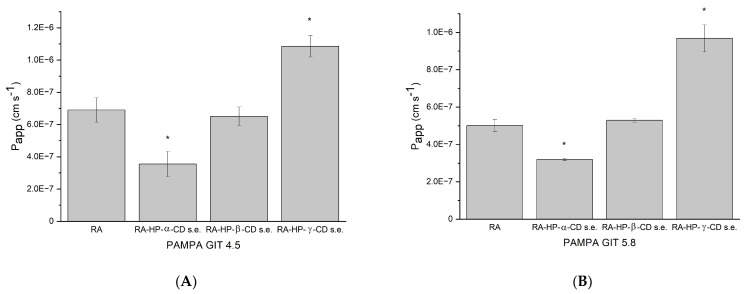
Apparent permeability coefficient values of RA and RA in binary systems prepared by the solvent evaporation method: RA–HP-α-CD s.e., RA–HP-β-CD s.e., RA–HP-γ-CD s.e. in a pH of 4.5 (**A**) and in a pH of 5.8 (**B**). (*) indicates statistically significant differences, *p* < 0.05.

Permeability coefficient of the RA in pH 4.5 is 6.901 × 10^−7^ ± 7.619 × 10^−8^ cm/s, which is less than 1.0 × 10^−^^6^ cm/s, so it is considered as poorly permeable (Figure 8A) [26]. The interaction of RA with HP-α-CD decreased the permeability to 3.553 × 10^−7^ ± 7.785 × 10^−8^ cm/s, as did the formation of the HP-β-CD (to 6.510 × 10^−7^ ± 5.803 × 10^−8^ cm/s). Nevertheless, the permeability of RA in the RA–HP-γ-CD s.e. system increased significantly to 1.085 × 10^−6^ ± 6.605 × 10^−8^ cm/s. Thus, the RA might be regarded as well permeable.

The RA permeability coefficient in pH 5.8 was determined as 5.019 × 10^−7^ ± 3.309 × 10^−8^ cm/s, which means it is poorly soluble (Figure 8B). RA’s permeability in the RA–HP-α-CD s.e. system is decreased to 3.197 × 10^−7^ ± 6.650 × 10^−9^ cm/s, while the system with HP-β-CD increased RA permeability to 5.296 × 10^−7^ ± 1.045 × 10^−8^ cm/s. The greatest change was noticed in RA–HP-γ-CD s.e., where the permeability coefficient reached 9.680 × 10^−7^ ± 7.235 × 10^−8^ cm/s, which is statistically significant. Even though the rise in permeability is noticeable, it did not cross the 1.0 × 10^−6^ cm/s; subsequently, it was still poorly permeable.

The BBB permeability was determined only for the RA, as CDs do not enter the bloodstream. The apparent permeability coefficient was 1.013 × 10^−6^ ± 9.176 × 10^−8^ cm/s, which suggests that RA penetrates the BBB barrier slightly [27].

### 3.9. Antioxidant Activity

In the study of antioxidant activity, the RA turned out to be a strong antioxidant whose IC_50_ (concentration needed to decrease the initial radical concentration by 50%) in the DPPH study was 59.483 ± 0.041 µg/mL and for ABTS 102.578 ± 3.427 µg/mL (Table 2). The reference substance, Trolox, shows a lower antioxidant activity—IC_50_ values for DPPH—of 93.640 ± 1.072 µg/mL and for ABTS 120.188 ± 2.726 µg/mL. The results of the CUPRAC and FRAP studies show similar relationships. IC_0.5_ (concentration indicating 0.5 absorbance) for RA in CUPRAC was 13.677 ± 0.993 µg/mL and for Trolox 56.564 ± 0.664 µg/mL, while in FRAP RA the IC_0.5_ was 10.558 ± 0.203 µg/mL and for Trolox 41.941 ± 0.014 µg/mL.

The scavenging potential of the RA in the DPPH and ABTS methods increased significantly for the system with HP-γ-CD. The IC_50_ value in DPPH was lowered to 57.398 ± 0.762 µg/mL and in ABTS to 87.766 ± 1.802 µg/mL. The systems with HP-α-CD and HP-β-CD also decreased the IC_50_ value and, thus, increased the scavenging activity; however, the changes were not statistically significant. DPPH and ABTS assays determine the ability of the substance to scavenge radicals, which is related to hydrogen-donating ability [50,51].

RA at concentration 5.869 ± 0.140 mg/mL, which is the value of its water solubility, inhibited AChE and BChE; however, this value is below the IC_50_ (the concentration that inhibits enzymes by 50%). RA inhibited AChE by 36.876 ± 1.812% and BChE by 13.68 ± 0.519%. The increase in RA solubility due to its connections with CDs to 113.027 mg/mL, 179.840 mg/mL, and 194.354 mg/mL using HP-α-CD, HP-β-CD, and HP-γ-CD, respectively, is significantly above the IC_50_, which is for AChE 8.983 ± 0.162 mg/mL and for BChE 14.865 ± 0.358 mg/mL, and enabled the achievement of maximum enzyme inhibition. The RA tyrosinase IC_50_ is 1.591 ± 0.077 mg/mL, thus, achieving 50% inhibition of the enzyme within RA’s water solubility.

## 4. Discussion

The RA shows a wide range of biological activities from antioxidant, anti-inflammatory, hepatoprotective, neuroprotective, and photoprotective to antidiabetic and antimicrobial properties [52,53]. The RA is well tolerable in healthy humans, and the administration of a single dose of *M. officinalis* extract comprising 500 mg of RA was assessed to be harmless [10]. The limitations of its preventive and therapeutic potential are its physicochemical properties such as slight solubility and poor permeability. This study aimed to overcome these obstacles by building RA systems with CDs. Water RA solubility at room temperature was assessed as 5.869 ± 0.140 mg/mL. The preparation of RA systems with CDs resulted in an efficient increase in solubility; the most extraordinary outcomes were obtained for RA–HP-γ-CD s.e., where there was a 33-fold increase to 194.354 ± 1.847 mg/mL. Hazim et al. designed a study where the supercritical carbon dioxide extraction of *Orthosiphon stamineus* leaves with the addition of ethanol was performed, and the optimization of the process to correlate the solubility of RA in supercritical carbon dioxide [54]. The greatest solubility was obtained in 80 °C and 10 MPa with 2.004 mg of RA/L solvent. In Sethi et al.’s study, RA was assessed to be turbid in water in a solubility study, while RA complex with phosphatidylcholine and phyto-vesicles resulted in water solubility in a visual study [55]. In a phase solubility study, the solubility of RA with HP-β-CD and M-βCD was increased 3.33 and 3.47-fold, respectively [16].

RA is unstable in gastric acidic pH, which is present in the fasted stomach [23]; thus, it might be beneficial not to administer it on an empty stomach. After food intake, gastrointestinal pH values vary, gastric emptying is delayed, bile flow is stimulated, and the splanchnic blood flow is increased [10]. All of the changes might occur to a different extent among different people, depending on their health and diet. In Noguchi-Shinohara et al.’s study, the food intake impacted the absorption of RA, and the area under the curve (AUC) of RA was increased [10]. Considering the above-mentioned results, dissolution and permeability studies were carried out in pH values resembling GIT-fed conditions. In apparent solubility studies, the RA in a pH 4.5 RA was dissolved in 46.94 ± 2.32% in 15 min and 89.97 ± 1.79% in 45 min. RA in RA–HP-γ-CD s.e. dissolved RA at 60.20 ± 2.76% in 15 min and 84.52 ± 3.18% in 30 min. At pH 5.8, RA was dissolved at 37.97 ± 1.04% in 15 min; 81.48 ± 2.10% in 45 min, and 99.35 ± 1.27% in 90 min. RA in the RA–HP-γ-CD s.e system reached 66.53 ± 0.43% in the fifteenth minute and 98.277 ± 1.19% in the sixtieth minute and remained constant. In Marinho et al.’s release study at pH 7.0 (dialysis bag method) from free RA solution, 74% of RA was released within an hour; from uncoated niosomes and chitosan-coated vesicles loaded with RA, around 70% of drug released after 8 h [56]. RA from an eletrospun mat with Eudragit E100 with ethanolic oregano extract was released in 500 mL of medium in a pH of 1.5 at 37 ± 0.5 °C at the maximum level—78% after 10 min, and it remained at this level until the end of the study [57].

In the study on Caco-2 cell monolayers, transport determined that RA mostly permeates via paracellular diffusion, and the intestinal permeability was <1% of its intake volume [58]. The intestinal permeability of RA in *Thunbergia laurifolia* extract was determined as better than pure RA. Moreover, *P_app_* values were increased after deconjugation by β-glucuronidase/sulfatase enzymes [8]. Additionally, RA undergoes degradation within GIT. In the study of Bel-Rhlid et al., the hydrolysis of RA from rosemary extract was studied in an in vitro GI model [59]. RA was not hydrolyzed chemically under the conditions of the GI model or by secreted enzymatic activity. However, probiotic *L. johnsonii* strains addition resulted in the (> 90%) hydrolysis of RA. This study attempted to overcome poor RA GIT permeability, which was confirmed in the PAMPA study, with a permeability coefficient in pH 4.5—6.901 × 10^−7^ ± 7.619 × 10^−8^ cm/s. However, the permeability was significantly increased by systems formation with HP-γ-CD to 1.085 × 10^−6^ ± 6.605 × 10^−8^ cm/s reaching a good permeability level. In pH 5.8, permeability was also assessed as low—*P_app_* 5.019 × 10^−7^ ± 3.309 × 10^−8^, whilst after preparation of the systems with the same CD it was significantly enlarged to 9.680 × 10^−7^ ± 7.235 × 10^−8^ cm/s, almost reaching a good permeability value (1.0 × 10^−6^ cm/s).

During the connections of RA with CDs, hydrogen bonds between these molecules are formed, weakening RA intramolecular hydrogen bonds and subsequently increasing the hydrogen donation by the hydroxyl groups of RA. The stronger the interaction between system components, the greater the hydrogen donation of RA, as the covalent bonds between hydrogen and oxygen in the hydroxyl groups are weaker.

RA might play a vital role in the prevention of neurodegenerative diseases, as it has neuroprotective and neurodegenerative properties and impacts neuroinflammation in in vitro and in vivo studies [60,61,62,63]. It inhibits reactive oxygen species generation, inhibits enzymes such as AChE, BChE, lipoxygenase, and cyclooxygenase, and inhibits both extracellular (amyloid beta) and intracellular (tau) protein aggregation [64,65]. Subsequently, the authors decided to determine the impact of systems formation on antioxidant and neuroprotective activity on RA. The antioxidant activity of RA was increased in DPPH (IC_50_ from 59.483 ± 0.041 µg/mL to 57.398 ± 0.762); ABTS (IC50 from 102.578 ± 3.427 to µg/mL 87.766 ± 1.802 µg/mL); and FRAP (IC0.5 from 10.558 ± 0.203 µg/mL to 10.126 ± 0.132 µg/mL) assays, and the results were reported as prepared concentrations. Similar trends were reported in other studies. In Veras et al.’s study, RA complexes with HP-β-CD and M-β-CD that were obtained by freeze-drying assessed greater antioxidant activity than pure RA in the DPPH assay [16]. The ability to scavenge radicals studied by the DPPH assay was also increased after complexation of cuminaldehyde and isoeugenol with M-β-CD by the ultrasonication method, and after preparing vitamin-A acetate/cyclodextrin nanofibrous webs by electrospinning [47,66]. In another study, antioxidant potential in the CUPRAC assay was determined for RA complexes with α-CD, β-CD, HP-β-CD, HE-β-CD, and M-β-CD that were obtained by freeze-drying [14]. All systems showed better reductive potential than pure RA, and the greatest results were obtained for a complex with M-β-CD. Chitosan–RA conjugates exerted significantly greater antioxidant potential in the DPPH assay (EC_50_ = 2.8–3.7 μg/mL) than pure RA (EC_50_ = 10 μg/mL) [53].

In most cases, the increase in solubility of the complexed substance for antioxidant activity is not without significance. In the case of RA, the IC_50_ and IC_0.5_ values are well below the water solubility of pure RA; therefore, the effect on the antioxidant potential is due to the systems formation. Very high CD concentrations might decrease the antioxidant potential of antioxidants, as the inclusion-complexation and systems formation equilibria of antioxidants may predominate those of their oxidation [14]. Thus, in this study, the concentrations of CDs were suitably low and in the tested concentrations showed no antioxidant potential in any studies. CDs can affect the redox behavior of the polyphenol [14]. Subsequently, the antioxidant potential of RA in the systems was increased. The reducing potential of RA was mostly increased for RA–HP-γ-CD s.e., IC_0.5_ for CUPRAC 13.823 ± 0.027 µg/mL, and FRAP 10.126 ± 0.132 µg/mL, which is statistically significant.

CDs also increase the antioxidant activity of other biologically active compounds. Daidzein–CD–metal organic framework solid dispersion complexes prepared by the solvent evaporation method increased daidzein DPPH scavenging activity ~1.3 times [67]. In another study, DPPH radical-scavenging activity test indicated that the caffeic acid complexes with α-CD and with β-CD obtained by the ground mixture method had greater antioxidant activity than caffeic acid; additionally, greater results were obtained for the complex with α-CD [68]. The preparation of a nutraceutical curcumin–piperine–2-HP-β-CD system prepared by the kneading method increased the antioxidant properties of the components [17].

The antioxidant activity of RA will contribute to the reduction in oxidative stress, protecting cells’ structure and function and preventing excessive chronic inflammation, thus, being a neuroprotectant. Neurodegeneration might also be prevented by inhibiting enzymes such as acetylcholinesterase, butyrylcholinesterase, and tyrosinase among patients with dementia [69].

To the best of the authors’ knowledge, there are no studies on the influence of amorphous or crystal systems influencing RA’s activity on AChE, BChE, and tyrosinase activity. Therefore, this study examined how the increase in the RA solubility affects the activity against these enzymes, since CDs do not leave GIT and do not directly affect the enzymatic activity. From 36.876 ± 1.812% of inhibiting AChE and 13.68 ± 0.519% of inhibiting BChE at the concentration 5.869 ± 0.140 mg/mL (water RA solubility), it was possible to obtain maximum enzyme activity (plateau) for each RA–HP-α-CD s.e., RA–HP-β-CD s.e., and RA–HP-γ-CD s.e. system, where the IC_50_ for AChE was 8.983 ± 0.162 mg/mL and for BChE 14.865 ± 0.358 mg/mL. RA tyrosinase IC_50_ was determined within RA water solubility.

## 5. Conclusions

Rosmarinic acid is a phenolic acid with excellent pro-health potential, but it is limited by physicochemical properties, which this study aimed to overcome. Interactions between the RA with cyclodextrins (HP-α-CD, HP-β-CD, and HP-γ-CD), especially in the case of HP-γ-CD, resulted in an increase in solubility, permeability, antioxidant activity, and the inhibition of acetylcholinesterase and butyrylcholinesterase.

## Figures and Tables

**Table 1:** The energy of binding determined for different types of RA–CD systems. Due to the high conformational flexibility of both guest and host molecules, minor differences in determined energies for various poses, as well as the equivalency of different binding poses, the given energy ranges correspond to systems of similar structural geometries. #: 2-HP-CD^#^ containing all 2-HP groups substituted to the O_(2)_ hydroxyl oxygen atoms of CD (atom numbering in accordance with the IUPAC recommendations); *: 2-HP-CD* containing all 2HP groups substituted to the O_(6)_ hydroxyl oxygen atoms of the hydroxymethyl groups present in CD.

Guest Molecule:	Binding Energy [kJ/mol]	
	Substitution Pattern *	Substitution Pattern ^#^
2-HP-α-CD	−5.0–−4.8	−5.4–−5.2
2-HP-β-CD2-HP-γ-CD	−6.2–−5.9−6.5–−6.1	−7.2–−6.9−7.0–−6.9

**Table 2:** The results of the antioxidant activity of RA and the systems (assessed as prepared concentrations). (*) indicates statistically significant differences, *p* < 0.05.

	DPPH	ABTS	CUPRAC	FRAP
	IC_50_ (µg/mL)	IC_50_ (µg/mL)	IC_0.5_ (µg/mL)	IC_0.5_ (µg/mL)
RA	59.483 ± 0.041	102.578 ± 3.427	13.677 ± 0.993	10.558 ± 0.203
RA–HP-α-CD s.e.	59.796 ± 0.042	106.940 ± 3.479	14.140 ± 0.106	10.435 ± 0.216
RA–HP-β-CD s.e.	58.490 ± 0.884	98.028 ± 4.223	14.331 ± 0.136	10.316 ± 0.152
RA–HP-γ-CD s.e.	57.398 ± 0.762 *	87.766 ± 1.802 *	13.823 ± 0.027	10.126 ± 0.132 *
Trolox	93.640 ± 1.072	120.188 ± 2.726	56.564 ± 0.664	41.941 ± 0.014

## Data Availability

Data are available in a publicly accessible repository.

## Abbreviations

ABTS	2,2′-azino-bis(3-ethylbenzothiazoline-6-sulfonic acid)
AChE	acetylcholinesterase
ANOVA	one-way analysis of variance
ATCI	acetylthiocholine iodide
ATR-FTIR	Attenuated Total Reflectance Fourier Transform Infrared spectroscopy
AUC	area under the curve
BBB	Blood–brain barrier
BChE	butyrylcholinesterase
BCS	Biopharmaceutics Classification System
BTCI	butyrylthiocholine iodide
CD	cyclodextrin
CUPRAC	cupric reducing antioxidant capacity
DMSO	dimethyl sulfoxide
DPPH	2,2-diphenyl-1-picrylhydrazyl
DSC	differential scanning calorimetry
DTNB	5,5′-dithio-bis-(2-nitrobenzoic) acid
FRAP	ferric reducing antioxidant power
GIT	gastrointestinal tract
HE-β-CD	2-hydroxyethyl-β-cyclodextrin
HPLC	high-performance liquid chromatography
HP-β-CD	2-hydroxypropyl-β-cyclodextrin
LLOD	lower limit of detection
LLOQ	lower limit of quantification
M-β-CD	methyl-β-cyclodextrin
PAMPA	parallel artificial membrane permeability assay
RA	rosmarinic acid
RPM	rotations per minute
TNB	3-carboxy-4-nitrothiolate
TPTZ	2,4,6-tris(2-pyridyl)-1,3,5-triazine
XRPD	X-ray powder diffraction
